# GENDER AND RACE AS MODERATORS BETWEEN ACTIGRAPHY SLEEP AND INFLAMMATORY MARKERS

**DOI:** 10.1093/geroni/igae098.0249

**Published:** 2024-12-31

**Authors:** Yuqi Shen, Linying Ji, Claudette Kessler, Christopher Engeland, Orfeu Buxton, Alan Zonderman, Michele Evans, Alyssa Gamaldo

**Affiliations:** The Pennsylvania State University, University Park, Pennsylvania, United States; Montana State University, Bozeman, Montana, United States; The Pennsylvania State University, University Park, Pennsylvania, United States; The Pennsylvania State University, University Park, Pennsylvania, United States; The Pennsylvania State University, University Park, Pennsylvania, United States; National Institute on Aging, Baltimore, Maryland, United States; National Institute on Aging, Baltimore, Maryland, United States; Clemson University, Clemson, South Carolina, United States

## Abstract

This cross-sectional study investigated gender and race differences in the association between actigraphic sleep (mean levels and variability) and inflammatory markers (circulating interleukin [IL]-6, IL-10, tumor necrosis factor [TNF]-𝛼 and C-reactive protein [CRP]). The study included participants from the HANDLSleep study (n=99; 74% female, 54% Black adults; age range: 46-82). Within-person mean and standard deviation (iSD) of sleep were estimated using one week of wrist actigraphy (mean ± SD = 6.8 ± 0.5 day), including nighttime sleep time, 24-hr sleep time, wake after sleep onset, nighttime sleep midpoint (Timing), and sleep maintenance efficiency. Linear regression models were conducted including interaction terms (race*sleep; or sex*sleep) and covariates (age, WRAT3 literacy scores, poverty status). Results indicated that race significantly moderated the association between variability in sleep maintenance efficiency and circulating IL-6 (interaction term p =.002). Race-stratified analyses revealed that among White adults, greater variability in sleep maintenance efficiency was significantly associated with higher circulating IL-6 (b = 0.10, p =.003). No significant association was found among Black adults. Results also indicated that gender significantly moderated the relationship between mean level 24-hr total sleep time and circulating IL-6 (interaction term p =.001). Stratification analyses revealed that longer mean 24-hr total sleep time was associated with higher levels of IL-6 among men (b = 0.003, p =.008). No significant association was found among women. Consistent with prior studies, the current study observed associations between actigraphic sleep and inflammation primarily among White adults and among men.

